# The Role of Adipose Tissue Mesenchymal Stem Cells in Colonic Anastomosis Healing in Inflammatory Bowel Disease: Experimental Study in Rats

**DOI:** 10.3390/jcm12196336

**Published:** 2023-10-02

**Authors:** Georgios Ntampakis, Manousos-Georgios Pramateftakis, Orestis Ioannidis, Stefanos Bitsianis, Panagiotis Christidis, Savvas Symeonidis, Georgios Koliakos, Maria Karakota, Chrysanthi Bekiari, Anastasia Tsakona, Angeliki Cheva, Stamatios Aggelopoulos

**Affiliations:** 1 4th Department of General Surgery, Aristotle University of Thessaloniki, 54124 Thessaloniki, Greece; gntampak@auth.gr (G.N.);; 2 Laboratory of Biochemistry, Aristotle University of Thessaloniki, 54124 Thessaloniki, Greece; 3 Experimental and Research Center, Papageorgiou General Hospital of Thessaloniki, 56403 Thessaloniki, Greece; 4 Laboratory of Anatomy and Histology, Veterinary School, Aristotle University of Thessaloniki, 54124 Thessaloniki, Greece; 5 Pathology Department, Faculty of Medicine, Aristotle University of Thessaloniki, 54124 Thessaloniki, Greece

**Keywords:** adipose tissue mesenchymal stromal cells, inflammatory bowel disease, bowel anastomosis, anastomotic leak, colorectal surgery, dextran sodium sulfate

## Abstract

(1) Background: A surgical operation on an inflamed bowel is, diachronically, a challenge for the surgeon, especially for patients with inflammatory bowel disease. Adipose tissue-derived mesenchymal stromal cells are already in use in clinical settings for their anti-inflammatory properties. The rationale of the current study was to use AdMSCs in high-risk anastomoses to monitor if they attenuate inflammation and prevent anastomotic leak. (2) Methods: a total of 4 groups of rats were subjected to a surgical transection of the large intestine and primary anastomosis. In two groups, DSS 5% was administered for 7 days prior to the procedure, to induce acute intestinal inflammation. After the anastomosis, 5 × 10^6^ autologous AdMSCs or an acellular solution was injected locally. Macroscopic evaluation, bursting pressure, hydroxyproline, and inflammatory cytokine expression were the parameters measured on the 8th post-operative day. (3) Results: Significantly less intra-abdominal complications, higher bursting pressures, and a decrease in pro-inflammatory markers were found in the groups that received AdMSCs. No difference in VEGF expression was observed on the 8th post-operative day. (4) Conclusions: AdMSCs attenuate inflammation in cases of acutely inflamed anastomosis.

## 1. Introduction

Inflammatory bowel disease (IBD) is an immunological disease of the gastrointestinal tract which is caused by an uncontrollable immunological response of the body. There are two distinct clinical types of IBD, Crohn’s disease (CD) and ulcerative colitis (UC), the incidence of which is significantly high in Europe compared to the rest of the world [1]. Demographically, CD affects younger patients in their 20s or 30s, while UC is more common in the 3rd–4th decade of life, even though their presence in all age groups (0–90) is not excluded [2]. As an illness that primarily affects young populations, which constitute the working power of society, IBD has a significant socio-economic impact in the modern world. Therapeutic costs, absence from work, and emotional disturbances are a great burden for society and the individuals suffering from these diseases. In addition, these patients suffer from a low quality of life after a surgical procedure that might require an ostomy, as well as the high inpatient costs due to prolonged hospitalization and perioperative complications [3].

With regards to the phenotype of the disease, one third of patients with CD are diagnosed with terminal ileitis; in another third of the patients, the disease is localized in the colon only; and the final third of patients present with ileocolic disease. Approximately 10–20% of the patients present with perianal disease (abscesses, fistulae). Approximately one third of patients with UC present with inflammation below the rectosigmoid junction; another third present with a diseased colon up to the splenic flexure; and the final third of patients present with extensive colitis [2].

The use of biological factors in the therapeutic arsenal of IBD has resulted in less surgical operations, offering patients a better quality of life, with longer disease-free intervals. Nevertheless, complications requiring surgical intervention can still occur, leading to extended bowel resections and stomas [4]. The selection of the most appropriate operation for each individual patient is tailored according to the characteristics of the individual patient’s disease. In all disease phenotypes, the operations include limited or extended resections with or without diverting stoma creation, and complex procedures to restore the continuity of the gastrointestinal tract [5,6].

In the last few years, in addition to other therapeutic approaches that utilize biological agents, mesenchymal stromal cells (MSC) have been utilized as an adjunct therapy. MSCs are multipotent cells that are considered to have immunomodulatory and anti-inflammatory properties. These cells can be harvested from bone marrow, placenta, or adipose tissue and be used either systematically or locally [7].

Recent studies have aimed to investigate the metabolic and molecular pathways through which the MSCs affect the inflammatory response; it is thought that MSCs affect the inflammatory response by regulating the expression of certain inflammation-related cytokines and reprogramming M1 macrophages [8,9,10,11,12,13,14,15,16]. Small molecules derived from the MSCs, called exosomes, might affect intracellular communication by altering the inflammatory response. This constitutes another experimental field which already shows promising results in the pre-clinical studies [12,17,18,19,20,21,22,23,24,25,26].

Currently, the use of MSCs in extraluminal CD (fistulae, abscesses) shows good results in clinical trials regarding their efficacy and safety. In addition, the literature shows that adipose tissue-derived mesenchymal stromal cells (AdMSC) are the best choice for fistulae therapy in CD [27,28,29,30,31,32]. Other MSC clinical trials target the recurrence of CD and UC with the systematic administration of MSCs; this approach has promising results, as the meta-analysis of Dave et al. shows [33].

Only a few studies have been designed to show the efficacy of MSCs in high-risk gastrointestinal anastomoses, but the experimental protocols used, and the variables measured, are very diverse between the studies. Van de Putte et al. have shown that MSCs have the ability to attenuate inflammation caused by preoperative radiotherapy, and ameliorate the quality of the anastomosis [34]. Pascual et al. have shown increased medium bursting pressure in a high-risk anastomosis with biological stromal cell embedded sutures [35]. A few studies have shown reduced leak rates and increased medium bursting pressure with MSCs in the ischemic anastomosis model [36,37,38,39]. Alvarenga et al. have shown, using an experimental high-risk anastomosis model, that the use of AdMSCs resulted in reduced local complication rates, the attenuation of the inflammation, and reduced tissue damage, as well as the downregulation of pro-inflammatory cytokines [40].

The objective of the current study is to experimentally confirm that AdMSCs can be applied on an acutely inflamed colonic anastomosis and decrease the risk of anastomotic complications and its derivatives (dehiscence, leak, abscess formation, peritonitis, adhesions, and sepsis).

## 2. Materials and Methods

### 2.1. Ethics Statement

All animal manipulations were in line with the current national laws for experimentation (PD 56/2013). The license number for this experimental study is #810556(3269) and granted by the prefecture of Central Macedonia. The ethical committee of Aristotle University of Thessaloniki (license no. 07/2020/07.02.2020) co-signed the approval of the current study. Compliance with ARRIVE guidelines was ensured during the approval process [41].

### 2.2. Experimental Design

For the purpose of the experiment, young Wistar rats (Rattus norvegicus) were used, and were 10–14 weeks old, with an average weight of 250–300 g. The rats were bred in the Research and Experimental Centre of the Papageorgiou General Hospital of Thessaloniki (license no. EL54BIOsup43), where they were hosted throughout the entire experimental process. The rats were living in pairs in their cages, with access to standard chaw and water ad libitum. Room temperature was stable at 22 °C and humidity was between 55% and 65%, while the cycle of light (12 h of light and 12 h of darkness) was maintained with an automatic switch.

Twelve rats received dextran sodium sulfate (DSS) 5% with water ad libitum to induce acute colitis [42]. The rest of the animals drank water without DSS.

After power analysis, 24 rats were used and were equally assigned randomly to the following experimental groups. Group A (Op): laparotomy with colonic anastomosis only. Group B (Op + AdMSCs): laparotomy with colonic anastomosis and local injection of 5 *×* 10^6^ AdMSCs in 70 μL power buffer saline (PBS) [34,43,44]. Group C (Op + DSS + Sal): DSS 5%, laparotomy with colonic anastomosis, and local injection of 70 μL power buffer saline (PBS). Group D (Op + DSS + AdMSC): DSS 5%, laparotomy with colonic anastomosis, and local injection of 5 *×* 10^6^ AdMSCs in 70 μL PBS.

All the animals were sacrificed on the 8th post-operative day according to local euthanasia protocols (CO_2_ cage).

After euthanasia, all animals were subjected to laparotomy. The abdominal cavity was then assessed, and a macroscopic complication score was calculated. After careful dissection, a 2.5 cm specimen was retrieved, and bursting pressure was measured. Finally, the specimen was split into two pieces, one for hydroxyproline measurement and the other for real-time PCR to measure IL-6, TNF-a, and VEGF.

### 2.3. Adipose Tissue-Derived Mesenchymal Stromal Cells

Fat tissue was washed with normal saline after harvesting from the rats’ right inguinal fold and cut into smaller pieces. Subsequently, it was processed with collagenase type I (0.5 mg/mL) in 37 °C for an hour. After homogenization of the tissue, the mesenchymal stem cell layer was isolated with centrifuge (2900 rpm, 20 min). Finally, the cell sediment was diluted in power buffer saline (PBS) to achieve a cellular solution of 5 *×* 10^6^ cells/mL^.^. The adipose-derived mesenchymal stromal cells were isolated according to the existing standardized protocol of the Laboratory of Biochemistry of our department. The AdMSCs were positive for CD44 and CD90 markers [45]. The primers used are mentioned in Appendix A Table A2.

### 2.4. Colitis Protocol

**Figure A1:**
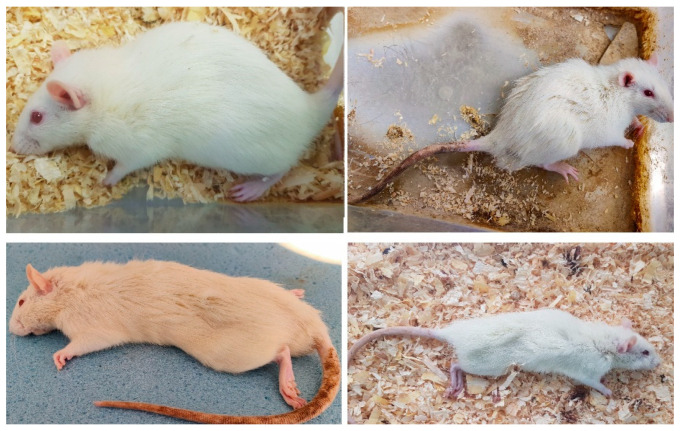
The first figure depicts the rat prior to DSS administration. The rest show their state after induction of colitis. Weight loss, hair erection, decreased mobility, and signs of neglected self-hygiene are noted.

**Figure A2:**
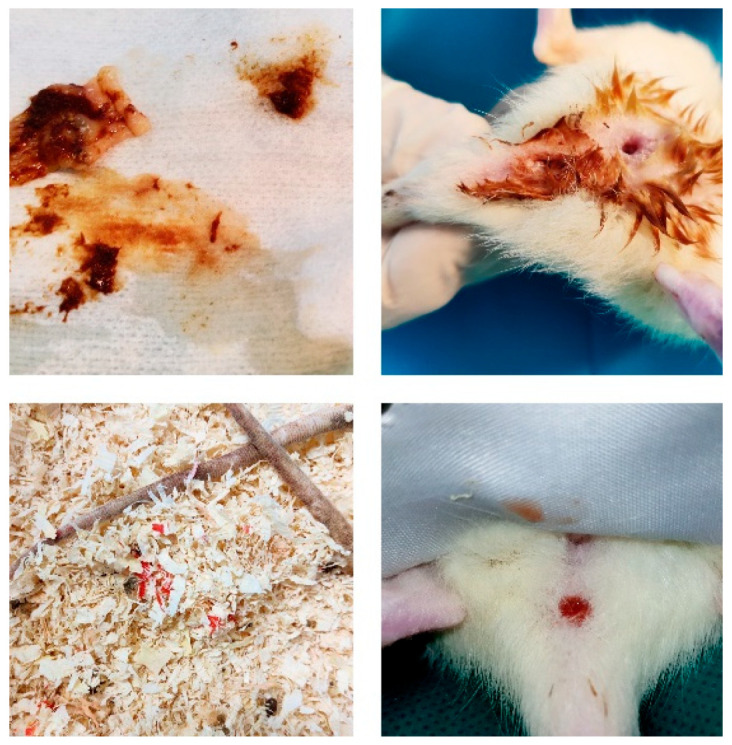
Mucous diarrhea, edematous anal mucosa, and blood in the stool after DSS administration.

**Figure A3:**
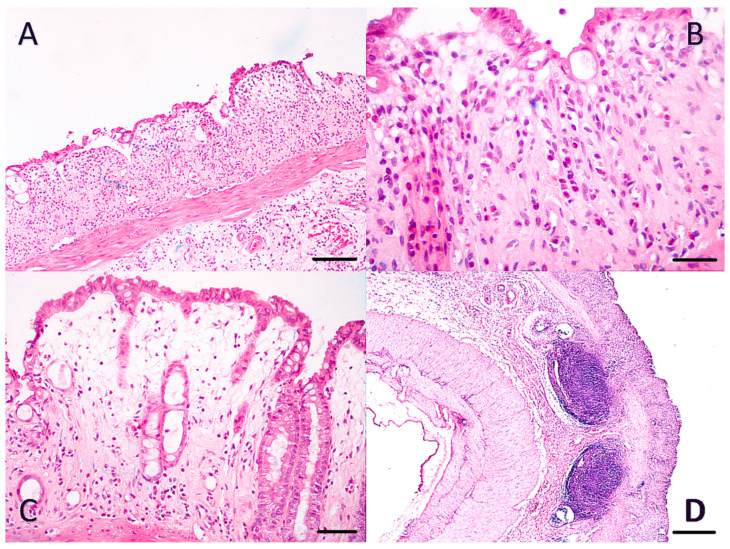
Histological confirmation of DSS colitis. H&E colon sections. ((**A**): ×100/scale bar 100 μm, (**B**): ×400/scale bar 20 μm, (**C**): ×200/scale bar 50 μm, (**D**): ×100/scale bar 100 μm). Figure A3 (**A**–**C**): Ulceration of the mucosa, inflammatory infiltration of mucosa, and submucosa from neutrophils. Obliteration of crypts and cryptic glands, as well as goblet cells. Loss of villous height. (**D**): Enlarged submucosal lymph nodes.

Dextran sodium sulfate (DSS) 5% 40 kPa was used for the induction of acute colitis in rats (Dextran Sulfate 40 Sodium Salt; AppliChem GmbH, Darmstadt, Germany) [46]. The chemical was added to the rats’ water, and access to it was allowed ad libitum. The rats were observed clinically for 7 days. Loss of weight, diarrhea, bloody stool, general decline, anal inflammation, erected hair, and signs of self-neglect (soiled tails) were indicative of active colitis in the rats. The above findings were confirmed histologically in control animals that received DSS, as shown in Figure A1, Figure A2 and Figure A3.

### 2.5. Operative Procedure

The rats were anesthetized prior to the procedure, with an intraperitoneal administration of 50 mg/kg ketamine and 5 mg/kg xylazine. Hair removal was performed with a hair clipper and the abdomen was prepared with povidone iodine solution.

**Figure A4:**
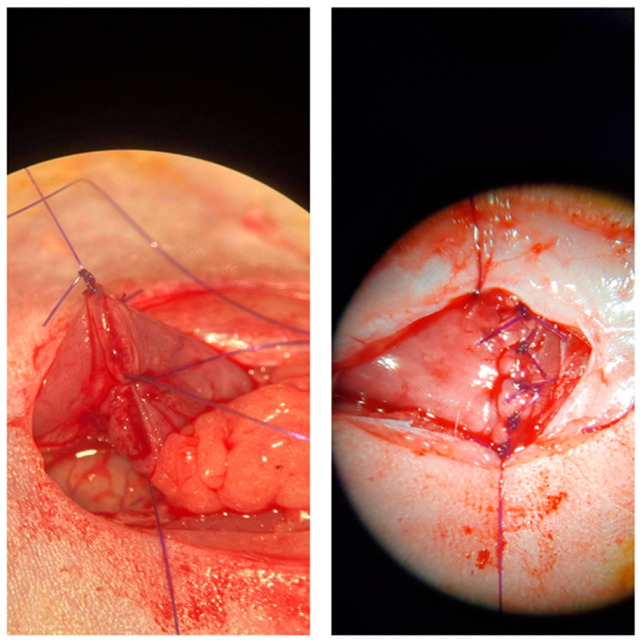
Anastomosis creation under the microscope.

A midline 3 cm laparotomy was performed, and the large bowel was identified on the left of the abdominal cavity. A transection of the descending colon was performed, just above the pubic symphysis, taking care to preserve the vascularization. Following the transection, in group 1 an end-to-end primary anastomosis with a 5/0 polydioxanone (PDS) suture was performed, with interrupted stitches with the use of a microscope. In groups B and D, 5 *×* 10^6^ AdMSCs in 70 μL PBS were injected in the bowel ends prior to anastomosis; in group C, 70 μL PBS was injected. After the anastomosis was completed, the bowel was returned to the abdominal cavity, and 10 mL of saline was used to wash the abdominal cavity. The surgical wound was closed with a 3/0 polyglactine suture, as shown in Figure A4.

After the operation, the rat was placed in an appropriately heated cage to recover and was allowed to consume water and food freely.

Seven days after the initial operation, all animals were sacrificed with use of a CO_2_ cage according to local euthanasia protocols. A midline laparotomy was performed, and the abdomen was assessed macroscopically. After that, a 2.5 cm segment of the bowel containing the anastomosis was resected, and bursting strength was measured. Following the measurement of bursting strength, the specimen was frozen with the use of liquid CO_2_ and was sent for biochemical measurements (hydroxyproline and real-time PCR).

### 2.6. Macroscopic Assessment

For the clinical assessment of the anastomosis, the anastomotic complication score was used, as proposed by Bosmans et al. [47], for the standardization of the clinical description of the experimental anastomosis outcomes (Table 1).

### 2.7. Bursting Pressure

**Figure A5:**
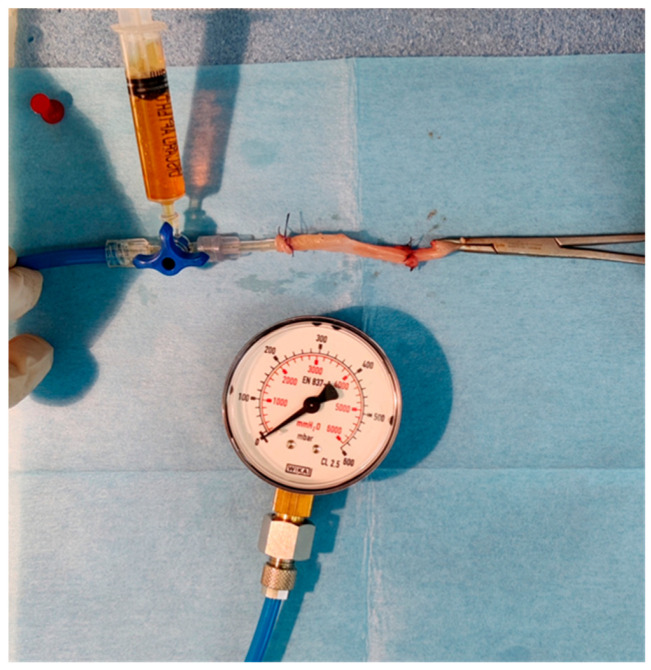
Device for measuring anastomotic bursting strength.

The bursting pressure of the specimen was measured ex-situ, with the device depicted in Figure A5 of the Appendix A [48,49]. It consisted of a simple manometer, a tube connected to a three-way canula, and a syringe containing dyed water connected to the three-way. The specimen was fixed with a purse string to the tube and the free edge was clamped.

The dyed water was then infused slowly inside the specimen and the procedure was recorded with a camera to ensure an accurate recording. The pressure under which the anastomosis burst constituted the bursting pressure and was logged.

### 2.8. Hydroxyproline

Part of the resected bowel was sent for hydroxyproline measurement. All specimens were dried in cold air and homogenized before the procedure. A hydroxyproline concentration was estimated with the use of a spectrophotometry with a wavelength of 550 nm, after preparation with certain solutions (Table A1).

### 2.9. Real Time-Polymerase Chain Reaction

For the measurement of inflammatory cytokines (IL-6, TNFa, VEGF), a NucleoSpin RNA kit (Macherey-Nagel, Düren, Germany) was used according to the manufacturer’s instructions. For the quantitative measurement of the cytokines, a One-Step qRT PCR kit (KAPABIOSYSTEMS, Wilmington, MA, USA) was used. The primers used can be found in Table A2.

### 2.10. Statistical Analysis

The measured variables were checked for the normality of their distribution by the Shapiro–Wilk test. Normally distributed continuous variables were expressed by the arithmetic mean ± standard deviation (mean ± SD), while continuous variables with a non-parametric distribution were expressed by the median and interquadrant range (median, IQR). Qualitative variables, categorical or ordinal, were presented as numbers and percentages per 100. The confidence interval was set at 95% which means that the differences between the groups were considered statistically significant when *p* < 0.05. To compare the independent variables in the two study groups, the Mann–Whitney U test was used. Non-parametric tests were preferred due to the small sample size. The statistical analysis of the results was performed using the statistical program Jamovi 1.6.18.0. All of the descriptive statistics can be found in Appendix B.

## 3. Results

### 3.1. AdMSCs Macroscopically Attenuate Intra-Abdominal Complications

**Figure 1:**
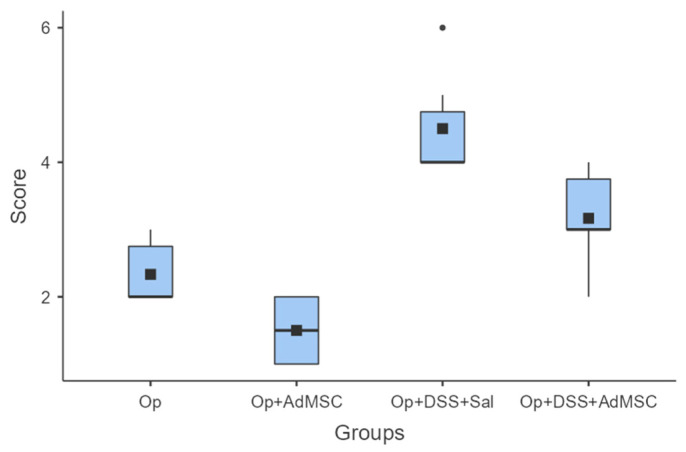
Macroscopic evaluation. Comparisons: A vs. B: *p* = 0.038, C vs. D: *p* = 0.02, B vs. D: *p* = 0.008.

Macroscopic evaluations of the abdominal cavities showed less intra-abdominal adhesions, colonic distention, and abscess formation. For groups A (operation only) and B (operation and AdMSCs), minimal complications were found, mainly adhesions to fat or other organs; group B, which received the AdMSCs, proved to have significantly less adhesions than group A (*p* = 0.038). As for the groups with acute inflammation, groups C and D, small abscesses, colonic dilatation, and other signs of intra-abdominal contamination were found, but again they were significantly more likely in group C, which did not receive AdMSCs. Group D had moderate complications compared to group C (*p* = 0.02); however, more significant morbidity was found in Group D compared to group B (*p* = 0.008). These results are illustrated in Figure 1 and Table A3.

### 3.2. Bursting Strength Is Significantly Higher in Groups with AdMSC

**Figure 2:**
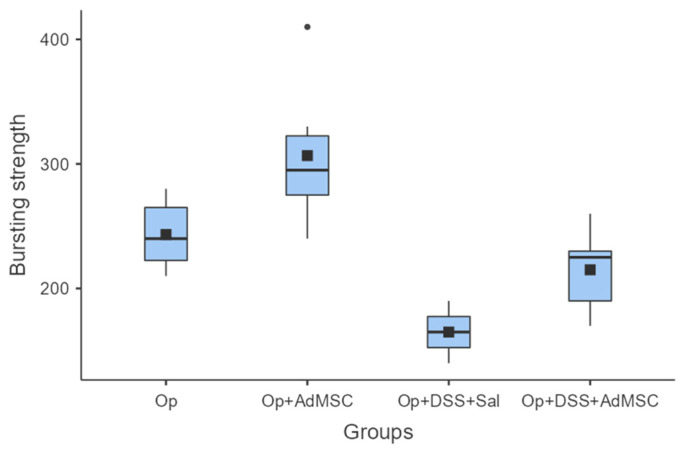
Bursting strength. Comparisons: A vs. B: *p* = 0.037, C vs. D: *p* = 0.03, B vs. D: *p* = 0.008.

It seems that the groups that received AdMSCs (B and D) had a relatively higher bursting strength than their counterparts. The mean bursting pressures are lower in groups with inflammation, but the mean pressures are significantly higher in groups that received AdMSCs compared to those that did not. Also, it appears that the AdMSCs applied to the anastomosis which was subjected to a surgical strike had a significantly higher bursting pressure (*p* = 0.037) (Figure 2, Table A4).

### 3.3. Anastomoses with AdMSCs Had More Collagen Deposition

**Figure 3:**
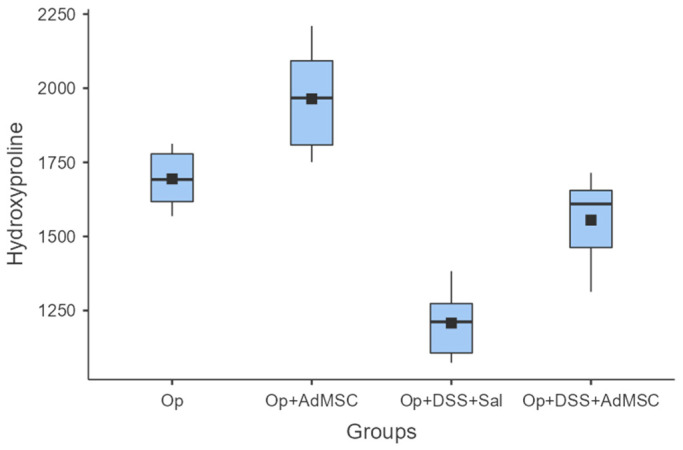
Hydroxyproline. Comparisons: A vs. B: *p* = 0.041, C vs. D: *p* = 0.004, B vs. D: *p* = 0.002.

Enhanced anastomotic healing was found in anastomoses with AdMSCs, as indicated by higher mean hydroxyproline concentrations. The highest mean hydroxyproline concentration was found in Group B, which was significantly higher compared to Group A (*p* = 0.041). Inflammation reduced the collagen deposition in the anastomosis, and therefore hindered healing, but the AdMSCs reverse this effect, as shown from the comparison of groups C and D (*p* = 0.004) (Figure 3, Table A5).

### 3.4. Pro-Inflammatory Cytokine Expression Is Downregulated in Groups That Received AdMSC

**Figure 4:**
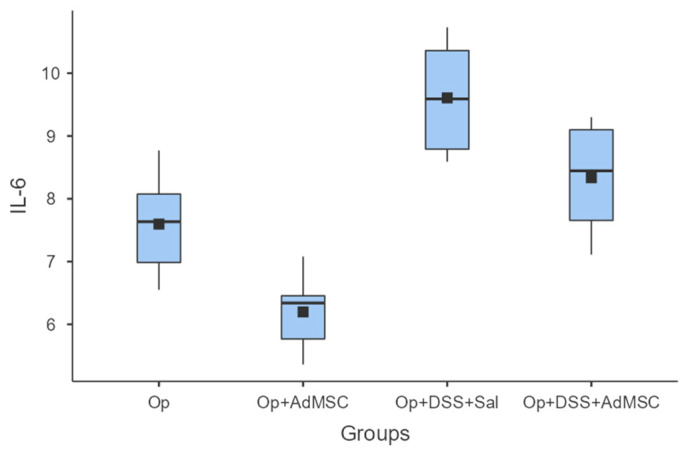
IL-6. Comparisons: A vs. B: *p* = 0.009, C vs. D: *p* = 0.065, B vs. D: *p* = 0.002.

**Figure 5:**
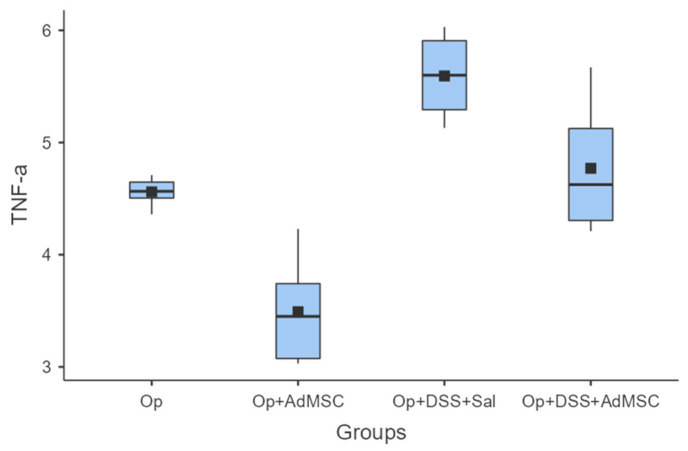
TNF-a. Comparisons: A vs. B: *p* = 0.002, C vs. D: *p* = 0.026, B vs. D: *p* = 0.004.

The mRNAs of TNF-a and IL-6 were over-expressed in the groups that received DSS; it seems that the AdMSCs helped to downregulate the expression of these genes in the groups in which it was administered. Even in group B, the pro-inflammatory cytokines were significantly reduced compared to the control (Group A). These results are illustrated in Figure 4 and Figure 5 and Table A6 and Table A7.

### 3.5. No Difference in Neo-Vascularization of the Anastomosis

**Figure 6:**
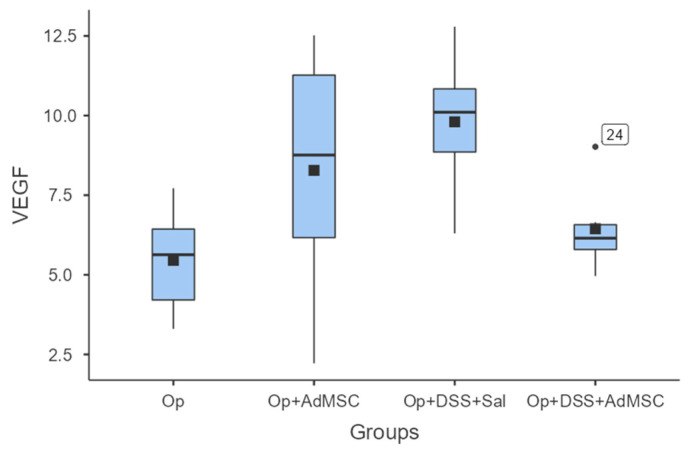
VEGF. Comparisons: A vs. B: *p* = 0.18, C vs. D: *p* = 0.065, B vs. D: *p* = 0.31.

With regards to neo-vascularization, we failed to see any significant differences between the groups. Although the mean values of VEGF were slightly higher in groups that received AdMSCs, there was no statistically significant difference between the respective groups. There was also a lower mean VEGF in group D compared to group B; therefore, the mean VEGF expression was lower in the group with acutely inflamed bowels, as shown in Figure 6 and Table A8.

## 4. Discussion

This study demonstrates that AdMSCs can be administered to colorectal anastomoses and attenuate inflammation. We showed that in the groups that received AdMSCs, bursting pressure was higher, local pro-inflammatory markers were not overexpressed, and anastomotic healing was enhanced, as shown by higher hydroxyproline levels.

The fact that inflammation impedes anastomotic healing is already a well-known fact in surgical practice, and is once again proved in our study, given that morbidity was higher in groups with acute inflammation. AdMSC administration reduced anastomotic-related morbidity, as shown by the macroscopic evaluation score, and by the lower rate of abscess formation, anastomotic dehiscence, and fecal peritonitis. There were no rat deaths in the groups that received AdMSCs, and there were fewer adhesions than the groups that did not receive AdMSCs. The low morbidity in groups that received AdMSCs has been found in similar research projects [39,40,50]. Yoo et al. reported significantly lower rates of infectious complications, strictures, and ulceration, but failed to show any significant difference in anastomotic leaks and adhesions in their ischemic anastomosis model [38].

Bursting strength was measured to be the highest in the groups that received AdMSCs in comparison to the groups that did not. Inflammation reduced the bursting strength of the anastomosis despite the administration of AdMSCs in group D, but bursting pressures were significantly higher compared to group C. Overall, the highest mean anastomotic pressure (mean, 307) was found in group B without the inflammation, with administration of AdMSCs [36,37,38]. In our study, the pressure measurements were measured ex-vivo so that inevitable local adhesions did not interfere with the bursting pressure and so we could gain a more objective appreciation of the anastomosis quality [35].

Hydroxyproline levels are a good marker of collagen accumulation in the anastomosis, but, in our view, its use as a marker of the quality of the anastomosis is oversimplified, because as far as we know, the anastomosis undergoes remodeling, which is mediated by cytokines like TGF-β, which induces the differentiation of fibroblasts to myofibroblasts [40,51,52]. In the remodeling phase, immature type III collagen is substituted by the more mature type I, which makes the anastomosis more durable [53]. Since our sampling takes place on the 8th post-operative day, we would argue that this period coincides with the proliferative phase of healing, during which we would expect an increased deposition of collagen in the anastomosis, which was demonstrated. Collagen was found to be higher in the groups that received AdMSCs [36,37,38].

Pro-inflammatory cytokines TNF-a and IL-6 were found to be significantly reduced in the groups that received AdMSCs and were following the same pattern as pro-inflammatory cytokines. We know that both these cytokines play an integral role in haemostasis/the inflammatory phase of anastomotic healing and are produced by M1 macrophages [54,55,56,57]. One of the mechanisms of action of AdMSCs is believed to be polarization of the M1 macrophage phenotype to the M2 type, which is believed to attenuate inflammation [34]. The M2 phenotype pro-inflammatory cytokines are downregulated and the cytokines involved in fibroblast proliferation and differentiation are increased. Gonzalez et al. administered AdMSCs to rats with colitis and sepsis. They have shown that AdMSCs had an anti-inflammatory effect by decreasing TNFa, IL6, and other proinflammatory cytokines, as well as increasing the expression of IL-10, thus downregulating the Th1 mediated inflammatory response, which is similar both in DSS colitis and Crohn’s disease [58].

Another important component of granulomatous tissue formation in anastomoses is neo-vascularization, which plays a very important role in anastomosis viability. This is induced by the increased secretion of the vascular endothelial factor (VEGF). In studies with models of ischemic colitis, the VEGF was increased in groups that received bone marrow derived MSCs (BmMCSs) [36,37,59]. Similar results have been produced by Van de Putte et al. using their radiation-induced colitis protocol [34]. To our knowledge, our study is the first to investigate VEGF expression using an experimental colitis model. It is shown that VEGF expression tends to be increased in all groups compared to the control, but there is no significant difference between the groups. This could be a result of the sampling timing occurring early in the healing process.

Although mesenchymal cells have already been used in clinical trials with good results, we are still at a quite premature stage of understanding the complex mechanisms by which they work, react with other cells, and regulate inflammation. It is still too early to interpret these results in humans, as the standardization of experimental procedures, and more in-vivo and in-vitro experiments are needed. Most of the protocols are designed on the assumption of an ischemic rather than inflamed anastomosis; therefore, more experiments are needed with this type of high-risk anastomosis. There is still no universal agreement on the concentrations of cells that are sufficient to augment anastomotic healing; more experiments on this subject need to be performed in the future.

Our findings are in agreement with studies that have shown that administration on site can help the properties of AdMSCs work in a paracrine way on the organ target. Van de Putte et al. have shown that the local accumulation of MSCs was not significant at the side of the anastomosis weeks after the administration of therapy; therefore, it is speculated that the therapeutic properties in the indigenous cells are mediated by molecules excreted by MSCs [34]. Pascual et al. used sutures coated with MSCs and showed that the cells were homogenously distributed in the anastomoses and resulted in more durable anastomoses with fewer local adhesions [35]. Adas et al. conducted similar studies by administering MSC therapy both systemically and locally. They found that locally transplanted MSCs resulted in accelerated healing and attenuated inflammation for ischemic bowel anastomoses, whereas there was no improvement in inflammation when MSCs were administered systemically [36,37]. Alvarenga et al. and Castelo-Branco et al. have similarly shown that the systemic administration of MSCs failed to reach the organ target and attenuate inflammation, as compared to the intraperitoneal administration of MSCs. In their latest work, they instilled MSCs locally, which resulted in the attenuation of the inflammation by the downregulation of proinflammatory cytokines, the upregulation of anti-inflammatory cytokines, and a decrease in the expression of metalloproteases [40,60]. Lee et al. have reported that intravenously injected MSCs were found to be trapped in the lungs [61]. Also, Yu et al. have reported no therapeutic effect when they injected the secretome of MSCs intravenously, as compared to applying the therapy locally with the use of fibrin glue as a medium [62].

In terms of administration, we have found that directly administering MSCs on the anastomosis site worked in producing these results. This is in agreement with studies that have shown that the administration of AdMSCs on the site of the anastomosis can help the properties of the AdMSCs work in a paracrine way on the organ target [34,35,36,40,50]. Most of the related studies agree that by using a parenteral administration method, MSCs hardly ever reach the organ target [34,62]. Further studies are needed to reveal whether mesenchymal cell properties can be amplified by using a different medium of application. Yu et al. have proposed a novel cell free therapy by using fibrin glue as a medium. They have demonstrated that it is possible to deliver the healing properties of MSCs by administering their secretome with fibrin glue, enabling the slow release of healing and growth factors for up to 10 days in rats with ischemic anastomoses [62].

This study proves that AdMSC therapy is feasible and promising and could potentially be translated into human studies in the future; however, there is still more work to be done, as biological responses can vary between different species. There are still unanswered questions regarding the minimal dosage of MSCs that will have the optimal effect in the healing of anastomoses, as well as whether there are agents or mediums of application that could enhance the therapeutic properties of MSCs. One of the potential drawbacks of MSC therapy could be the hyperexcretion of growth factors that could theoretically lead to carcinogenesis. After proving that MSCs are effective at attenuating inflammation, the next step would be to prove their safety, before applying the treatment in human trials.

Key to the therapeutic properties of AdMSCs is the secretome by which they seem to regulate inflammation as well as enhance the healing properties of the cells; this might indicate future experimental directions. As indicated by the studies of Park et al. and Yu et al., using AdMSC secretome could be a way of using the properties of MSCs without using the actual cells [62,63]. More studies with secretome could potentially lead us to identify the molecule or the group of molecules produced by MSCs which have similar anti-inflammatory and regenerative properties as the cell culture of MSCs.

Nevertheless, regardless of the future findings and possible implementations in daily practice, MSC experiments help us to better understand and gain new insights into how anastomotic healing works.

## 5. Conclusions

In this study, we investigated the potential of adipose tissue-derived mesenchymal stromal cells (AdMSCs) for mitigating complications associated with high-risk anastomoses, particularly in the presence of acute intestinal inflammation. The results obtained reveal several significant outcomes. The administration of AdMSCs led to a notable reduction in intra-abdominal complications, including adhesions to fat and other organs, and significantly increased the bursting strength of anastomoses. Furthermore, AdMSCs promoted enhanced collagen deposition, which is indicative of enhanced healing in the early stages of the healing procedure, and downregulated the pro-inflammatory cytokines TNF-a and IL-6. Although AdMSCs had a potential positive effect on neo-vascularization, this difference was not statistically significant. These findings collectively support the clinical potential of AdMSCs in improving surgical outcomes and reducing inflammation in procedures with high-risk anastomoses, warranting further research and clinical investigation.

## Figures and Tables

**Table 1:** Anastomotic complication score as endorsed by Bosmans et al. Reprinted from [47].

Anastomotic Complication Score	
0	No adhesions or abnormalities
1	Adhesion to fat pad, clean anastomosis underneath
2	Adhesion to intestinal loop, abdominal wall or other organ
3	Anastomotic defect found underneath adhesion, no other abnormalities
4	Signs of possible contamination (e.g., small abscesses)
5	Clear anastomotic complication; free pus, obstruction, signs of peritonitis
6	Fecal peritonitis/Death due to peritonitis

## Data Availability

Data are available to any qualified researchers upon request to gntampak@auth.gr.

## Appendix A

**Table A1:** Solutions used for Hydroxyproline measurements.

#	Solution
1	Citric acid buffer pH = 6.5
2	50% k-propanol
3	Chloramine-T, 0.056 M
4	NaOH 10.125 N
5	Ehrlich 1 M reagent
6	Acetic acid 0.5N
7	Collagen standard solution 1 mg/mL

**Table A2:** Primers for RT-PCR.

	Ascending Primer (5′–3′)	Descending Primer (5′–3′)
TNF-a	TACTGAACTTCGG GGTGATCGGTCC	CAGCCTTGTCCCT TGAAGAGAACC
IL-6	CAAGAGACTT CCAGCCAGTTG	TTGCCGAGTAGAC CTCATAGTGACC
VEGF	CGCCTTGGCT TGTCACATC	GTCGGAGAGC AACGTCACTA
GAPDH	ACCACAGTC CATGCCATCAC	TCCACCACC CTGTTGCTGTA

## Appendix B

**Table A3:** Macroscopic evaluation.

Groups	Mean	Median	SD	Min	Max
A Op	2.33	2	0.516	2	3
B Op + AdMSC	1.50	1.5	0.548	1	2
C Op + DSS + Sal	4.50	4	0.837	4	6
D Op + DSS + AdMSC	3.17	3	0.753	2	4

**Table A4:** Bursting strength.

Groups	Mean	Median	SD	Min	Max
A Op	243	240	28.0	210	280
B Op + AdMSC	307	295	58.9	240	410
C Op + DSS + Sal	165	165	18.7	140	190
D Op + DSS + AdMSC	215	225	33.9	170	260

**Table A5:** Hydroxyproline.

Groups	Mean	Median	SD	Min	Max
A Op	1694	1692	103	1568	1813
B Op + AdMSC	1964	1967	186	1751	2210
C Op + DSS + Sal	1208	1212	120	1074	1383
D Op + DSS + AdMSC	1555	1609	155	1313	1715

**Table A6:** IL-6.

Groups	Mean	Median	SD	Min	Max
A Op	7.60	7.63	0.831	6.55	8.77
B Op + AdMSC	6.20	6.34	0.626	5.36	7.08
C Op + DSS + Sal	9.61	9.59	0.925	8.59	10.73
D Op + DSS + AdMSC	8.33	8.45	0.919	7.11	9.30

**Table A7:** TNF-a.

Groups	Mean	Median	SD	Min	Max
A Op	4.56	4.56	0.126	4.36	4.71
B Op + AdMSC	3.49	3.45	0.485	3.03	4.23
C Op + DSS + Sal	5.59	5.60	0.381	5.13	6.03
D Op + DSS + AdMSC	4.77	4.63	0.584	4.21	5.67

**Table A8:** VEGF.

Groups	Mean	Median	SD	Min	Max
A Op	5.45	5.63	1.67	3.30	7.72
B Op + AdMSC	8.28	8.76	3.91	2.22	12.52
C Op + DSS + Sal	8.94	8.89	2.62	5.30	12.79
D Op + DSS + AdMSC	6.10	6.14	2.03	3.84	9.54
